# Presence of* Rickettsia* Species in a Marginalized Area of Yucatan, Mexico

**DOI:** 10.1155/2018/7675828

**Published:** 2018-06-05

**Authors:** Gaspar Peniche-Lara, Bertha Jimenez-Delgadillo, Claudia Munoz-Zanzi, María Cárdenas-Marrufo, Carlos Pérez-Osorio, Juan Arias-León

**Affiliations:** ^1^Laboratorio de Enfermedades Infecciosas y Parasitarias, Facultad de Medicina, Universidad Autónoma de Yucatán, Mexico; ^2^Division of Environmental Health Sciences, School of Public Health, University of Minnesota, Minneapolis, MN, USA

## Abstract

In the state of Yucatan, Mexico, rickettsiosis has become a common vector-borne disease in the general population. Ectoparasite species such as* Rhipicephalus sanguineus *and* Amblyomma mixtum *have been identified as* Rickettsia* vectors in Yucatan by studies focused on the wild animal population in rural areas. There have been studies that have tried to determine the presence of* Rickettsia *species in ectoparasites collected in Yucatan, but these studies did not include marginalized areas, where living in close contact with domestic and peridomestic animals that carry ectoparasites is a high-risk factor for acquiring rickettsial infection or many other vector-borne diseases. We evaluated the vector diversity and the presence of* Rickettsia *species presence in the ectoparasite population that parasitizes domestic animals in a marginalized rural town of Yucatan, Mexico; we also evaluated the seroprevalence of rickettsial antibodies in the human population of this town in order to determine the prevalence of rickettsial infection. A total of 437 ectoparasites were collected from the study area. The tick specimens collected belonged to the species* Rhipicephalus sanguineus *(n=380, 49 positive),* Amblyomma mixtum *(n=3, 0 positive),* Ixodes affinis *(n=4, 0 positive),* Ctenocephalides felis *(n=33, 0 positive), and* Trichodectes canis *(n=17, 0 positive). Conventional polymerase chain reaction and sequencing were used to identify the DNA of* Rickettsia*. Six out of 354 (1.8%) serum samples were positive for antibody to* R. typhi*. The combination of low antibody titers and the presence of* Rickettsia* species infecting ectoparasite species found in the study area requires eco-epidemiological studies and the identification of potentially protective practices or habits.

## 1. Introduction

In the state of Yucatan, Mexico, rickettsiosis is the main vector-borne disease due to the high presence of tick and flea species in urban and suburban areas [1]. It is well known that rickettsiosis is commonly associated with poverty and rural areas [2]; however, in the state of Yucatan, most of the patients with rickettsiosis live in urban areas. Because of this, very little is known about the transmission dynamics of* Rickettsia *species in rural areas of Yucatan. A first approach to the study of these dynamics is to assess the seroprevalence of rickettsial antibodies in the population and to examine locally collected ectoparasites to determine the presence of* Rickettsia *species. This would help us know if the lower rate of rickettsial infection in the rural population of Yucatan could be due to a lower presence of* Rickettsia *species in rural areas or to practices and habits of the rural population that inhibit the life cycle of* Rickettsia *sp.

## 2. Materials and Methods

### 2.1. Study Site

The study was conducted in the Mayapan municipality (20°28′05′′N–89°12′50′′W), located southeast from Merida, the capital of Yucatan. It is considered one of the ten most marginalized municipalities in Yucatan, according to a report by the Consejo Nacional de Población (a government department that estimates the marginalization index in Mexico). The population of Mayapan work mainly in agriculture, hunting, and construction jobs in Merida; almost all the population speak both the Spanish and Mayan languages.

### 2.2. Collection of Ectoparasites

From June to July 2015, 188 houses were randomly visited and all domestic animals present in them (dogs, cats, horses, etc.) were examined, with previous authorization by the owner, looking for ectoparasites in body sites such as ears, shoulders, udder, and belly. The collected ectoparasites were pooled by individual host animal, preserved in ethanol (70%), and stored in our laboratory at -80°C. The collected ectoparasites were identified using recent entomological keys [3–6].

### 2.3. Sera

Serum was collected from the adult population of Mayapan who signed an informed consent form before collecting blood samples. The collected blood samples were allowed to clot at room temperature and then centrifuged; after separating the serum, it was stored at -20°C until further analysis.

### 2.4. Indirect Immunofluorescence

Two sets of 12-well slides were used for these experiments, one with* Rickettsia rickettsii* (*R. rickettsii*) Sheila Smith strain and the other with* Rickettsia typhi* (*R. typhi*) Wilmington strain. They were provided by Dr. Donald Bouyer from the Laboratory of Pathology of the University of Texas, Medical Branch. The collected sera samples were diluted (1:64) in PBS with 3% nonfat powdered milk, and 10*μ*L of diluted serum was added to each well of the antigen slides. The slides were then incubated for 30 min in a humid chamber. The slides were rinsed once and washed in PBS containing 0.05% Tween 20 for 10min. Fluorescein isothiocyanate-conjugated goat anti-human IgG, at an optimal working dilution of 1:40, was used as the secondary antibody. After incubation, the slides were rinsed once in PBS before mounting coverslips and observed using epifluorescence microscopy (Ultraviolet Microscope, Nikon, Inc.) at 400X magnification. Maximum titers were determined by examining serial 2-fold dilutions.

### 2.5. DNA Extraction

DNA samples were obtained from individual lice, fleas, and ticks. Before DNA extraction, the ectoparasites were washed twice with ethanol (70%) and then with sterile water and dried. DNA was extracted using ZR Tissue & Insect DNA MiniPrep™ (Zymo Research, USA) and stored at -20°C.

### 2.6. Detection of Rickettsia by PCR and Sequencing

The amplification of the extracted DNA was performed using the primers for the* Rickettsia *ARN 16S gene (fD1: AGAGTTTGATCCTGGCTCAG and Rc16S.452n: AACGTCATTATCTTCCTTGC primer pairs) and the* glt*A gene (RpCS.877p: GGGGGCCTGCTCACGGCGG and RpCS.1258n: ATTGCAAAAAGTACAGTGAACA primer pairs) [7] to amplify 426bp and 381bp gene fragments, respectively. Conventional polymerase chain reaction was performed using Thermo Scientific Maxima Hot Start PCR Master Mix (2X) (Thermo Scientific, USA). The obtained PCR products were purified using a Zymoclean™ Gel DNA Recovery Kit (Zymo Research, USA) and then direct-sequenced using an ABI PRISM™ 310 Genetic Analyzer (Applied Biosystems, USA). The obtained sequences were aligned using the BioEdit Sequence Alignment Editor v7.2.5. [8] and compared with those deposited in GenBank using Basic Local Alignment Search Tool (BLAST).

## 3. Results

### 3.1. Collection of Ectoparasites

A total of 437 ectoparasites were collected from 90 animals (63 dogs, 19 horses, and 18 cats) from the study area. The collected specimens of ticks belonged to the species* Rhipicephalus sanguineus *(*R. sanguineus*) (n=380),* Amblyomma mixtum *(*A. mixtum*) (n=3),* Ixodes affinis* (*I. affinis*) (n=4),* Ctenocephalides felis *(*C. felis*) (n=33), and* Trichodectes canis* (*T. canis*) (n=17) (Table 1).

### 3.2. Indirect Immunofluorescence

Six out of 354 (1.8%) serum samples were positive for antibody to* R. typhi*, with titers ranging from 1:64 to 1:256; however, no samples positive for antibodies to* R. rickettsii* were obtained. All but 1 of the subjects positive to exposure were female, with an age range of 30 to 76 years and an average age of 52 years. Of the positive subjects, five reported cohabiting with domestic animals such as dogs and cats, two reported the presence of rodents or rodent droppings in their houses, and none had been exposed to opossums.

### 3.3. Detection of Rickettsial Infection

A total of 49 (38 females, 10 males, and one nymph)* R. sanguineus* specimens tested positive for* Rickettsia DNA*. No* Rickettsia *DNA was identified in the other tick, flea, and mite specimens collected in the study site. The DNA consensus sequence obtained had a 100% homology (GenBank accession number KY328828) with the* R. rickettsii *species identified in previous studies on urban areas of Yucatan, 99% similarity to the* R. rickettsii* Morgan and R strains (KU323867.1, CP006010.1, CP006009.1), based on the ARN 16S PCR fragment sequenced, and 100% and 99% similarity (GenBank accession number KY328749) to the* R. rickettsii *strain identified in collected* Amblyomma parvum *and* R. sanguineus *ticks from Yucatan (KC469610.1, JX198506.1), based on the obtained* glt*A gene fragment.

## 4. Discussion

Important rickettsial diseases are mainly transmitted by tick species like Rocky Mountain spotted fever in the Americas (RMSF) and boutonneuse fever in the Mediterranean region and Africa [7, 9, 10]. The seroprevalence of rickettsial agents in the rural population studied here contrasts with previous results reported for Yucatan, in which 5.6% of the tested population had positive reactions to different rickettsial antigens [11]. That study included small towns and cities of Yucatan but no marginalized populations. In the study, it is interesting that this infection is less prevalent in a highly marginalized population. Current practices and habits of the rural population that prevent the transmission of infection from reservoirs of the disease might explain this lower prevalence. Some of these practices may include cohabitation with cats, which control the pests that harbor tick or flea species, or with dogs, which keep opossums away from the houses. Because these practices would not explain the exposure to* R. typhi*, since the flea vectors responsible for its transmission are harbored mainly by the population which is reduced by those practices, rodents and opossums, one might expect an increase in the risk of exposure to* R. felis*. Given that cats and dogs are usually infested with cat fleas, it is possible that the antibodies detected using* R. typhi* antigen are actually a cross-reaction with* R. felis*, but this requires further investigation.

The presence of* R. rickettsii *in* R. sanguineus *ticks is very interesting and contrasting. This is not the first report of the presence of this* Rickettsia *species in Yucatan, Mexico, it increases the possibility that* R. rickettsii* could have a homogeneous distribution, not only in the state of Yucatan but throughout the entire Yucatan Peninsula, using* R. sanguineus *and* A. mixtum *as a vector. In contrast, in Baja California, at the north of Mexico, outbreaks of Rocky Mountain spotted fever were associated with an increase in the presence of both* R. sanguineus *ticks infected with* Rickettsia* species and stray dogs [9, 12]. Further collaboration studies are required to evaluate and compare biological, ecological, and/or evolution factors that contribute to the increase of the possibility of* R. sanguineus* biting humans in north and south of Mexico. Other tick species (*A. mixtum*) infected by* Rickettsia *species such as* Rickettsia amblyommatis* have been identified by previous studies in the west coast of Mexico [13].* Amblyomma mixtum *is important because it frequently bites humans [14] and is considered as the preferred vector of* R. rickettsii *in other countries in Latin America, commonly Brazil [15–18].

Since the Rocky Mountain spotted fever (RMSF) is well known to aggressively affect humans and we found the presence of a tick species that frequently transmits RMSF, it is possible to say that the rural population of Yucatan must be at high risk of acquiring rickettsial infection; however, there must be other biological, ecological, or mainly social factors that decrease this risk and which should be analyzed from an eco-epidemiological point of view [19].

## Figures and Tables

**Table 1:** Data from collected ectoparasites.

Host	Ectoparasite	Life Stage	Sex	Total Collected	Total Positive
Dog	*Rhipicephalus sanguineus*	Adult	Female	249	38
Dog	*Rhipicephalus sanguineus*	Adult	Male	138	10
Dog	*Rhipicephalus sanguineus*	Nymph	-* *-	50	1
Horse	*Amblyomma mixtum*	Adult	Female	1	0
Dog	*Amblyomma mixtum*	Adult	Female	2	0
Cat	*Ixodes affinis*	Adult	Female	3	0
Cat	*Ixodes affinis*	Adult	Male	1	0
Dog	*Ctenocephalides felis*	Adult	-* *-	32	0
Cat	*Ctenocephalides felis*	Adult	-* *-	1	0
Dog	*Trichodectes canis*	Adult	-* *-	17	0
